# Food insecurity in later life: A food security framework to support preventative action

**DOI:** 10.1017/S0029665125102012

**Published:** 2025-11-19

**Authors:** Angela Dickinson, Claire Thompson

**Affiliations:** Centre for Public Health and Community Care, University of Hertfordshire, Hatfield, AL10 9AB; Food Inequalities and Health, Centre for Research in Public Health and Community Care, University of Hertfordshire, Hatfield, AL10 9AB

**Keywords:** Food insecurity, older adults, malnutrition, food security

## Abstract

Longer life expectancy and growing income inequality have prompted an increasing interest in understanding the impact of ageing on nutritional requirements in order to optimise intakes, increase the number of years lived in good health, and reduce morbidity and associated health and social care costs. Food insecurity reduces access to nutritious and healthy food. Understanding the evidence-base on the impacts of food insecurity and the maintenance of food security for older people is crucial to informing policy and intervention.

The increase in numbers of older people experiencing food insecurity is a public health emergency, and is associated with under and malnutrition. Food insecurity can be experienced at any stage of the life course, but has been more widely studied with families and children where poverty is a major driver. Food insecurity in later life has been less well explored by academics, but differs from that experienced in earlier years due to additional complexities, as physical and cognitive health amplify the impact of poverty. Additionally, factors which can appear to be relatively small in impact, can act in a cumulative way to push people towards food insecurity. This review will draw on research about older people’s food practices, contexts, and experiences in relation to food insecurity in later life, and offers a model of food insecurity that has the potential to guide focused public health efforts in order to support the older population to be food secure.

## Introduction

Vulnerabilities are differentially experienced by older people when compared to younger demographic groups. In order to minimise vulnerability at this life stage an understanding of the specific needs and traits of the older population is required. This includes identification of risk factors that lead to vulnerability; and implementation of effective programmes and policies ^(1)^. In this paper, we argue that experiencing food insecurity in later life exposes people to significant adversity and requires a range of approaches in order to respond to the diversity and complexity of food insecurity as experienced by the older population ^(1)^.

Food insecurity has adverse impacts both for those directly affected, as well as wider society as there are economic consequences, including increased use of health and social care services. The accumulation of threats to the UK food system has been described as a worsening ‘perfect storm’ ^(2)^. Ensuring that older households are food secure enables people to age well and is crucial to their health and well-being.

This review explores vulnerability in relation to food insecurity in later life. Food insecurity is defined as ‘the inability to consume an adequate quality or sufficient quantity of food in socially acceptable ways, or the uncertainty that one will be able to do so’ ^(3)^. This definition describes a multi-dimensional conceptual framework of FI that includes social and psychological aspects alongside nutritional elements.

### Prevalence of FI in older populations

Measuring prevalence of FI is important as a precursor to understanding the scale of the problem to determine appropriate action, with surveys being the main method to establish prevalence data. FI has only been measured in the UK in large representative surveys since 2016, when the Food Standards Agency (FSA) included the US Department of Agriculture’s (USDA) Adult Food Security Module ^(4)^ (as part of the *Food and You* Survey, since 2019 FI has been measured in the Family Resources Survey). In 2023 the *Food and You 2 survey* ^(5)^ found 92 % of older adults reported being food secure, with 9% of adults aged 65-79 living in households classified as experiencing low or very low FI, with figures of 6% for adults aged over 80 years. Low income, though an important factor, in itself did not fully account for FI, with age (older people being less likely to report being FI than younger adults) and home ownership (those who rented their homes were more likely to be FI than home-owners) being important factors. Over 5 million people aged 60+ in the US are estimated to be food insecure ^(6)^ with FI estimated to have increased over 45% in the past 20 years, with a prevalence of 14% of the older population in the US ^(7)^. In Canada, around 2.4% of older people are estimated to be moderately or severely food insecure, with income being the main predictor ^(8)^.

Differences in measurement tools used both within and between countries make it difficult to compare cross-national prevalence levels of FI. For example, in Australia, a single item ‘run out of food’ was being used to estimate FI in national surveys. However, recently the 18-item Household Food Security Survey Module (HFSSM) has been used alongside this. Tucher *et al* ^(9)^ argue that those measuring FI in later life tend to focus on financial considerations, and developed a broader based summary indicator that included three domains: functional, social support, and financial limitations using this in a secondary analysis of a large data set in the US.

#### Precarity and Food Insecurity in later life

Food insecurity (FI) in the older population has similarities and differences to that of younger demographic groups and households ^(10)^. However, despite knowledge that vulnerability to food insecurity has a differential impact on the older population, it is an area that has attracted very little academic attention ^(6)^. Although there is an acknowledgement that Pensioner Age Households (PAH) experience FI differently ^(11)^, most studies have focused on children, young people and families. Older people experience risk factors in common with people of all ages (for example inadequate income, and the price and availability of food) however, there are significant differences which affect the capacity of older people to respond to the challenges posed by FI. These include having smaller social networks to draw support from ^(12)^, social isolation and loneliness ^(13)^, and the increased likelihood of them having at least one long-term condition, mobility issues or sensory loss. Risk factors can be present singularly or in combination to these make the process of accessing food challenging ^(14)^.

Food insecurity is affected by a range of social determinants of health among older adults ^(7)^. Social determinants of health (SDH) include e.g. education, housing quality, income, and the food environment. Income is important as it influences other determinants, including well-being, mental health and food practices ^(15)^. Poverty affects a range of health outcomes and behaviours, leading to increased precarity of those affected. Grenier ^(16)^ argues that the ‘concept of precarity draws attention to insecurities in the context of global economic and social change, including unwanted risks, and the costly hazards of contemporary life that result from globalization, neoliberalization and declining social protection’. This leaves ‘older people suffering from unequal access to material goods, and the absence of public forms of care and support’ (^(16)^ p 73-74). The higher cost of healthier food means that those experiencing food insecurity are more likely to consume cheaper and more energy dense foods. Those with long terms conditions may be less able to prepare healthy foods in their own homes, increasing reliance on purchasing ready meals, takeaway and other less nutritious foods ^(17) (18)^.

### Threats to food insecurity in later life

Threats to food insecurity operate at different levels to influence how FI is experienced; including macro (e.g. national level where government decision-making and policies, such as state pension/benefit levels can affect individual experiences), meso (community level, where local government decisions and third sector and community organisations operate) and micro (household) level threats affect pensioner age adults (PAH).

#### Macro level threats to food insecurity

The Covid-19 pandemic revealed the extent of FI in communities ^(19)^. Since the UK emerged from the pandemic, the country has been exposed to a number of macro-level disruptions that have affected national food security, including those resulting from the exit of the UK from the EU. These disruptions have been further amplified by the co-existence of geopolitical challenges including the Russian invasion of Ukraine (with consequent trade disruption); and the climate emergency (extremes of weather leading to reductions in crop yields), all of which has contributed to the cost of living crisis affecting the availability and cost of food ^(20)^. More recently, cyber-attacks targeted at food retailers have further disrupted food supply chains at community levels i.e. reducing food availability on supermarket shelves ^(21)^. The cumulative impact of these shocks is food supply chain disruption, increasing food and energy costs and rising inflation that erodes the value of household incomes. Pensions, savings and benefits have been affected by inflation, thus pushing more households into poverty ^(22)^. Recently, Age UK estimated that 2 million older households do not have sufficient finances to cover essential spending, with 26% of those surveyed spending less on food shopping than previously ^(23)^. The cost-of-living crisis has resulted in cheaper, staple foods that form the basis of low-income diets such as pasta and rice rising in price disproportionately to other goods, with increases of up to 50% for some items ^(24)^. The recent policy indecision as to whether and how to means test the winter fuel allowance for older people in the UK has introduced further precarity. It initially meant (winter 2024-25) that only those in receipt of pension credit could receive this benefit ^(25)^. All these crises along with over a decade of austerity has weakened the ability of the public service resources of the State and the National Health Service to adequately respond to and prevent FI ^(20)^.

#### Meso level challenges to food insecurity

Threats to food insecurity operating at a meso (community) level include lack of resources available in local communities or the absence of age friendly food environments, such as poor provision for older people (and others experiencing disabilities) by supermarkets ^(26–28)^.

During the pandemic, many lunch clubs and other groups that traditionally provided food, social and well-being opportunities for older people and had operated for prolonged periods of time, were forced to close during lockdown with many not re-opening ^(29, 30)^. Access to services such as public transport, particularly in rural communities, adds additional barriers ^(31, 32)^. Increasing distance to food shops (over 0.25 miles) and living in a disadvantaged neighbourhood increase risk of FI, a situation that many older people who retire to coastal and rural communities find themselves in. However, community social cohesion is protective against FI and can mitigate these challenges ^(33)^. Recent years have seen a number of fluctuations in threats operating at the household level, which were particularly exposed during the COVID-19 pandemic, where there was a rapid deployment of a number of responses that aimed to support people with food security, particularly during the first lockdown ^(34)^. Some responses were made at a national level- e.g. food boxes supplied to those deemed to be particularly vulnerable to viral infection ^(27)^. However, most responses were made at a local community level (meso) to support older people to access food. Established local organisations were better able to rapidly respond with many already supporting vulnerable people in their communities e.g. volunteer support with shopping through initiatives such as Food Train Scotland ^(35)^. Those providing food and nutrition support to older people reported a substantial increase in referrals to their services and demand for them, especially meals-on-wheels ^(36)^. Restrictions put in place to contain the spread of the virus meant that less support around food from family members was available to older people. Community groups and services had to address this. Those organisations who were already delivering prepared food and meals to older people started to combine their deliveries with welfare checks ^(27)^. However, many of these interventions have not been sustained. The pandemic impacted on services that traditionally supported older people with access to food, as many services changed, were newly set up or closed.

#### Micro level challenges to food insecurity

Older households can be vulnerable to food insecurity through changes being experienced at the household (micro) level. These disproportionate effects can result from factors such car access ^(32)^, social isolation, bereavement ^(37)^, non-inclusive design ^(38)^ and lack of digital skills. Prior to the pandemic, 11% of people aged over 65 experienced difficulties getting to a local shop and 12% struggled to access a supermarket. Mobility challenges affected 18% of people aged 60-69 years and 38% people aged over 70, with more than 2 million people aged 65+ living with sight loss, making shopping more difficult ^(14)^. An Age UK survey found that since the pandemic, 43% of those aged 60+ (around 416,000 people) who were struggling with preparing and cooking food before lockdown were finding these activities more difficult. Around one in ten (1.45 million people) were finding it difficult to walk short distances outside, when this was not a problem for them before ^(39)^. These changes, if not addressed, could result in an increased demand for social care. Older people are especially vulnerable to food insecurity when they experience a change in personal health and at major life transition points, such as bereavement or following discharge from hospital when food needs are not always considered ^(40)^.

### The health consequences of FI in later life

Food insecurity has consequences for health and well-being, impacting on a wide range of health indicators and is associated with increased use of healthcare ^(41)^. An estimated 300,000 in the UK require assistance to prepare a hot meal ^(42, 43)^. Threats can often accumulate to push people towards food insecurity ^(44)^.

In older households, food insecurity increases the likelihood of under and malnutrition, and associated morbidity and mortality ^(45)^. Older adults who are food insecure have been found to have poorer nutritional intakes (both macro and micro-nutrients) which is associated with increased risk of many chronic diseases, such as Type 2 diabetes mellitus, cardiovascular disease, frailty, falls and mental ill health including depression and cognitive impairment ^(6, 7, 46, 47)^. In addition, FI is linked to risk of food borne illnesses as people are more inclined to engage in risky behaviour, such as using food beyond the use by date ^(48)^.

### Food insecurity and Malnutrition

Food insecurity is associated with malnutrition in older people ^(49)^ affecting over 1.3 million people aged over 65 in the UK ^(50)^. Malnutrition had an estimated cost to the UK economy of £23.5 billion in 2015 ^(51)^ equating to around 15% of the health and social care budget ^(52)^. Older people accounted for half of this spend ^(53)^. A systematic review and meta-analysis comparing prevalence of malnutrition risk in hospital and community settings found that in the UK, 43% were malnourished in hospital settings, with no UK data reported for prevalence in community settings ^(54)^. The authors found levels of community malnutrition across Europe of 28% in hospitals and 8.5% in community settings. A meta-analysis of 240 studies involving 113,967 subjects (where malnutrition risk was based on the use of Mini Nutritional Assessment (MNA)) found rates of malnutrition were 2.1% and risk of malnutrition was 23.4 % ^(55)^ in European studies.

Nutritional status is associated with the health status of older people, including poor mobility ^(32)^, chemosensory losses ^(56, 57)^, difficulty swallowing, changes to mental health including cognitive impairment ^(58)^. Social circumstances such as loneliness ^(59)^ and lack of economic resources ^(60)^ are associated with malnutrition in older age.

### Models of food security/insecurity in later life

A number of studies have attempted to develop models to either enable understanding of FI or predict or determine the best way to measure or quantify food insecurity. Typically, these draw on statistical modelling, based on analysis of large data sets. The statistical models incorporate pre-determined risk factors to estimate prevalence of FI and the associations with a range of social, demographic, geographic, and economic factors collected as part of the survey.

A number of studies have used the social ecological model (SEM) to underpin their analysis to inform understandings of food insecurity in later life, e.g. Goldberg & Mawn ^(46)^ used this model to determine the antecedents of food insecurity in later life using a secondary analysis of US NHANES data. The SEM focuses on 5 spheres; the intrapersonal, interpersonal, organisational, community and public policy. Tucher *et al* ^(9)^ used the same model to develop a summary indicator used to undertake the secondary analysis. They found three domains to be important to FI: functional, social support, and financial limitations. Lee *et al* ^(11)^ used the SEM model to explore publicly available data, and identified 13 risk factors for FI, finding that after financial issues, having a disability was the main indicator, with distance to the nearest grocery store the least important, and that taking these additional factors into account, levels of FI in the older population was higher than generally reported.

Steiner *et al* ^(61)^ aimed to develop a predictive model for individuals with FI, as determined using a single item added to the Medicare annual wellness survey ‘Do you always have enough money to buy the food you need?’ However, this question was not tested against any validated instruments. They found that food insecurity was associated with minority race or ethnicity and Medicaid insurance coverage, as well as other social determinants of health such as low education and social isolation. However, their predictive model could not identify which individuals could benefit from referral to community-based services providing food support to help address FI. All of these studies were undertaken in the US where a number of large datasets are available to researchers.

Two models were identified that used qualitative data of the experiences of older people. Wolfe *et al*. ^(10)^ interviewed 53 older people in the US, on 2 occasions, 6 months apart to explore FI from older people’s perspective. They note that although the U.S. Household Food Security Survey Module is a national measure of food insecurity, it is based on research in families with children. The aim was to improve the survey design when administered to PAH. They tested 14 new items in telephone interviews and recommended the following new items were included: “couldn't afford right foods for health”, “couldn't get the food I needed” and “unable to prepare”.

Dickinson, *et al*. ^(62)^ developed a *Food Security Framework* (FSF) from a qualitative ethnographic study of 25 PAH where in-depth data was collected using a range of visual methods (video go along shopping trips, photographs, observations, diaries and informal interviews). The FSF was adapted for FI from a four-domain model of vulnerability drawn from environmental sciences focusing on natural disasters ^(63)^. The FSF includes social determinants of health and incorporates the factors that protect people from or move them towards vulnerability to FI (threats, such as bereavement, visual impairments) as well as Coping Capacity (assets, such as social networks and access to services such as lunch clubs) that protects people from becoming vulnerable to food insecurity. One novel feature of this model is that it recognises that factors that could be thought to be relatively minor (such as access to clean toilet facilities), can accumulate to threaten FI (cumulative trivia ^(64)^).

Recognition that factors can accumulate and amplify each other, could help account for the difficulty found by the studies identified by O'Keeffe *et al* ^(65)^ when attempting to determine the modifiable factors contributing to malnutrition. The FSF has been further elaborated in a study on meals on wheels ^(66)^. Additionally, the model demonstrates that vulnerability to FI is reversable, and it enables those aiming to implement public health interventions to target interventions that support the avoidance of food insecurity. Interventions can be focused on avoiding or reducing threats (e.g. avoiding developing policies that adversely affect PAH), or focusing on providing interventions that can strengthen the older person’s coping capacity (e.g. providing opportunities for social eating). Further work is needed to test the public health intervention aspects of the model in a prospective study.

## Discussion

Understanding the lives of older people, including their financial circumstances and use of welfare benefits, social networks and the impact of illness and disability is essential to understanding risk factors that lead to vulnerability to food insecurity at a household level. Risk factors for FI operate at macro, meso and micro levels of society, and therefore solutions are also required across these same levels. Food insecurity is a complex state requiring long term, sustainable solutions developed with a range of actors, including government, NGOs, business and civil society to address low income, rising food prices and welfare reform ^(67)^. Canada has lower levels of FI than comparable countries such as the US. Canada introduced a Poverty Reduction Act in 2019 which has led to increasing income support to PAH to increase their economic security, though women and people of particular ethnic groups are more likely to experience low income providing an example of addressing poverty and thus FI at a macro level ^(68)^.

The pandemic exposed both the vulnerabilities of many older adults and the patchy health and social care provision that left them unable to respond to a crisis of the scale presented by the pandemic. Lang *et al* ^(69)^ highlight the need for better preparedness of the state and outlines 7 steps for action needed for civil food resilience. The pandemic highlighted the benefits offered by charities and social enterprises who were able to fill the gaps, providing and leveraging social capital and connection between older households and their neighbourhoods to support food security^(34)^.

### What can be done to address FI in pension age households?

Traditionally a number of services have supported PAH to maintain their food security and continue to live in their own homes. However, there is no one solution to FI that works for everyone. The All-Party Parliamentary Group on Hunger, ^(70)^ noted a number of examples of innovative community organisations doing important work to protect people from malnutrition which had the potential to be scaled up across the UK. Interventions included Food Train Scotland which offers a shopping and delivery service to older people in Scotland ^(35)^, meals on wheels (MoW) services and lunch clubs which in addition to providing a hot meal, provide opportunities for social interaction and activities.

Despite an increasing body of research demonstrating the health, nutritional and social benefits of MoW services ^(66, 71–75)^, their provision has been in catastrophic decline. The decline has been accelerated by the austerity measures pursued by the UK government from 2010 to 2024 resulting in the decommissioning of MoW services ^(76)^. MoW provision in London was found to be inadequate before the pandemic ^(77)^. There is currently no statutory duty for local authorities to provide MoW services, leaving MoW services vulnerable to funding shortfalls, with little consideration of the evidence, thought of the adverse impacts on service users, or the increased costs to other parts of the care economy.

Other community meal provision includes lunch clubs, day care, coffee mornings and more recently ‘warm spaces’ all of which provide important opportunities for social interaction and help build social and community networks ^(78)^.

Currently, UK adult social care is under pressure with care providers struggling to recruit and retain staff. A 2022 report from the Association of Directors of Adult Social Services noted that ‘A significant number of people are not getting the essential care and support that they need, leading to increases in unmet and under met need. The increasing volume and complexity of need is far outstripping the capacity to meet it.’ ^(79)^ p17). Care workers are under increasing pressure to deliver care in unrealistic time frames. If older people feel that their home care is rushed, it affects the care relationship, they feel unable to ask for the help they need, thus affecting their ability to feel safe in their own homes ^(80)^.

In addition to activities focusing on prevention, many studies recommend a need for a concerted effort to address malnutrition in older people, this includes the need to prevent, recognise and manage malnutrition once recognised ^(81)^. Health and social care practitioners could do more to recognise, support and address FI ^(82)^. The European Society Parental and Enteral Nutrition (ESPEN 2019) guidelines ^(58)^ on clinical nutrition and hydration in geriatrics make a number of recommendations including recommending that all older persons are routinely screened for malnutrition in order to identify an existing risk early. They recommend screening takes place in general practice at least every 12 months.

A number of challenges to nutritional screening have been identified including psychosocial barriers such as loneliness and reluctance to be screened. They argued that interventions aiming to implement screen-and-treat approaches to malnutrition in primary care should consider barriers that both patients and healthcare professionals may face ^(83)^. Other authors argue that increased attention to malnutrition screening of older adults in settings outside of hospital is needed ^(84)^. A study in Austria explored the impact of volunteers delivering home-based physical training along with nutritional advice for those who were frail/prefrail older people. They found not only that this approach helped tackle malnutrition, but social support on its own was also effective ^(85)^.

### Community food sector

Despite the high numbers of older people experiencing under and malnutrition, relatively few older people seek support from food banks. However, recently the numbers of older people reliant on food aid, such as food banks and food pantries is increasing ^(86)^. Anecdotal data indicate an increase in malnutrition in the community with people presenting to GPs with fatigue and tiredness due to malnutrition ^(87)^. A 2023 survey from the Independent Food Aid Network found that food banks were seeing an increase in older people needing help ^(88)^. Trussell have reported that that although the prevalence of poverty in pensioner age households (PAH) is lower than households with working age adults, the rates of poverty have increased to 18%, with 6% of support provided by food banks goes to PAHs. This equates to a 345% increase since 2018/19 ^(89)^. A number of barriers that prevent PAH from accessing support from food aid include stigma and pride ^(43, 82, 90)^. Structural factors such as difficulty accessing food banks, carrying food home and lack of technological expertise also limit use.

Tailored services, delivery options, wrap around support, and outreach need to be embedded in food aid provision for older households. We need better understanding of food insecurity in the older population, including well designed studies of how best to intervene to support older people to remain food secure, i.e. what interventions work for who and when.

## Conclusions

The increase in numbers of older people experiencing food insecurity should be considered a public health emergency, particularly due to the association with malnutrition and the consequences both for the health of the individual but also the cost to the health and social care economy. Older people experience a differential vulnerability to FI due to them experiencing additional challenges including physical and cognitive health issues which amplify the impact of poverty and act in a cumulative way to push people towards food insecurity. This review offers a model of food insecurity that has the potential to guide focused public health efforts and further research on interventions that can support the older population to be food secure.
